# Editorial on Special Issue “Skeletal Radiology”

**DOI:** 10.3390/diagnostics13142396

**Published:** 2023-07-18

**Authors:** Atefe Pooyan, Ehsan Alipour, Arash Azhideh, Majid Chalian

**Affiliations:** Division of Musculoskeletal Imaging and Intervention, Department of Radiology, University of Washington, Seattle, WA 98105, USA; atefe@uw.edu (A.P.);

## 1. Introduction

Musculoskeletal (MSK) disorders are among the top five contributors to disability-adjusted life years (DALYs) worldwide [1]. These disorders include a wide range of conditions, spanning from fractures, sports injuries, rheumatoid arthritis, and musculoskeletal pain syndromes to those mostly affecting older age groups like osteoarthritis and osteoporosis. MSK conditions often present diagnostic challenges due to overlapping symptoms, complex anatomical aspects, and the variety of potential causes [2]. Imaging plays a pivotal role in diagnosing and guiding treatment for these disorders. In the past decade, the MSK radiology field has witnessed significant progress driven by breakthroughs in artificial intelligence (AI), the development of high-resolution equipment and novel imaging sequences, and the adoption of innovative multidisciplinary approaches. This Special Issue on skeletal radiology brings together a diverse collection of articles that showcase the latest advancements in this field. From evaluating flatfoot to exploring emerging targets for osteoarthritis treatment, the included studies provide valuable insights into diagnostic accuracy and the expanding applications of skeletal radiology techniques. This editorial aims to highlight the significance of these articles and their contributions to the field of skeletal radiology.

### 1.1. Diagnostic Accuracy in Musculoskeletal Radiology

Several articles in this Special Issue delve into the diagnostic accuracy of radiological measurements in different musculoskeletal conditions. Tagliafico et al. investigated the reliability of the Myeloma Spine and Bone Damage Score (MSBDS) in assessing myeloma-related spine and bone damage using whole-body computed tomography (WBCT) [3]. This multicenter study emphasizes the need for standardized scoring systems to accurately evaluate myeloma-related skeletal complications. The researchers propose a consensus-based, semiquantitative scoring system based on CT data on multiple myeloma, which demonstrated a substantial level of agreement among readers of varying experience levels, highlighting its potential for widespread use. Another noteworthy study focuses on the diagnostic challenges of the osteonecrosis of the femoral head [4]. This disorder afflicts at least 30,000 Americans each year and can be undetectable in radiographs during the early stages [5]. Cardin-Pereda et.al. highlight the significance of a multidisciplinary approach in enhancing diagnostic accuracy. By considering various radiological modalities and incorporating clinical expertise, this study provides valuable insights into optimizing the diagnostic pathway for patients with osteonecrosis. Various radiologic measures are available for evaluating flatfoot; however, the accuracy of these measures is still unknown [6]. Thus, one of our studies provided a cross-sectional evaluation and differentiation of flatfoot that explores the sensitivity and specificity of six different measures, namely, arch angle, calcaneal pitch, talar-first metatarsal angle, lateral talar angle, talar inclination angle, and navicular index, in diagnosing flatfoot deformity [6]. This study will help to improve the accuracy of diagnosing flatfoot, leading to more appropriate treatment strategies. In another study, the accuracy of critical shoulder angle (CSA) and the acromial index in predicting supraspinatus tendinopathy was explored, emphasizing the significance of radiological measurements in assessing shoulder pathologies [7]. Critical shoulder angle, defined as the angle between the plane of the glenoid and the most lateral border of the acromion process, has been reported to be a predictor of several shoulder pathologies [8]. However, its association with supraspinatus tendinopathy had not yet been investigated. This retrospective case-control study shows that CSA can also predict supraspinatus tendinopathy.

### 1.2. Novel Applications and Techniques in Skeletal Radiology

This Special Issue also explores the application of novel techniques to skeletal radiology. New sequences are expanding diagnostic capabilities, and AI-based techniques are revolutionizing the field via enhancing image interpretation and improving image acquisition protocols. Despite being introduced some time ago, the utilization of relatively new MRI sequences like DWI in skeletal radiology has been limited due to their susceptibility to artifacts and inhomogeneity [9]. However, with the advancement of rapid acquisition techniques, researchers are now actively investigating the effectiveness of DWI in detecting and evaluating musculoskeletal pathologies. In their review study on advanced imaging techniques for assessing multiple myeloma, Torkian et al. explored the emerging role of MRI sequences such as DWI, intravoxel incoherent motion (IVIM), and positron emission tomography–magnetic resonance imaging (PET-MRI) in the evaluation of multiple myeloma [10]. The growing role of AI in medical imaging is exemplified in the study by Hong et al. [11]. In their study on lumbar spine computed tomography to magnetic resonance imaging (CT to MRI) synthesis, the researchers employed a generative adversarial network (GAN) model to generate synthesized images. They assessed the accuracy of these synthesized images through a visual Turing test, demonstrating the potential of GANs in bridging the gap between CT and MRI. Significant progress has also been achieved in the discovery of potential therapeutic approaches. For example, Talaie et. al. reviewed the emerging targets for osteoarthritis treatment [12]. They focused on exploring new investigational methods, such as genicular artery embolization (GAE), to identify neo-vessels as potential targets for the treatment of osteoarthritis, emphasizing the importance of understanding the mechanisms of inflammation, neovascularization, and joint remodeling in the pathogenesis of osteoarthritis.

### 1.3. Clinical Applications and Reviews

In addition to diagnostic accuracy and advancements in skeletal radiology, this Special Issue features studies focusing on specific clinical applications. The diagnosis of systemic disorders, characterized by their wide-ranging impact on different organs, significantly relies on the field of skeletal radiology. Due to the non-specific nature of their clinical presentations and the presence of overlapping features, familiarity with the radiologic findings of these disorders is crucial. Radiologic and dermatologic presentations of systemic disorders, the visualization of dialysis-related amyloid arthropathy via 18F-FDG PET-CT scans, and radiographic findings of inflammatory arthritis and mimics in the hands are the topics of the studies focusing on systemic disorders in this issue [13,14,15]. The other included reviews focus on pathologies presenting in specific anatomic locations. The imaging of the temporomandibular joint and postoperative findings of common foot and ankle surgeries are the topics of two of the included image-rich reviews that discuss common skeletal findings [16,17]. Notably, one article delves into posteromedial lesions of the chest wall, while the other focuses on reviewing grade 1 and 2 chondrosarcomas of the chest wall, providing crucial insights into accurate diagnosis and treatment planning for these complex cases [18,19].

## 2. Conclusions

This Special Issue on skeletal radiology encompasses a comprehensive array of articles that contribute to the advancement of this dynamic field. From studies on diagnostic accuracy to emerging applications and clinical reviews, the included articles show the progress and potential of skeletal radiology in improving patient care. The field of skeletal radiology offers promising prospects. Ongoing discussions focus on leveraging emerging technologies to enhance diagnostic capabilities and treatment planning. However, challenges persist, such as the need for standardized protocols, the addressal of data quality and privacy concerns, and the optimization of the integration of artificial intelligence. Further research is required to explore the potential of novel imaging techniques, including advanced MRI sequences and functional imaging modalities, in providing valuable insights into skeletal pathologies. The corresponding evolving technology and imaging techniques have the potential to revolutionize the practice of skeletal radiology, enabling more accurate diagnoses, personalized treatment approaches, and improved patient outcomes.
